# Occurrence of Acrylamide in Italian Baked Products and Dietary Exposure Assessment

**DOI:** 10.3390/molecules25184156

**Published:** 2020-09-11

**Authors:** Francesco Esposito, Salvatore Velotto, Teresa Rea, Tommaso Stasi, Teresa Cirillo

**Affiliations:** 1 Department of Public Health, University of Naples “Federico II”, via Sergio Pansini, 5–80131 Naples, Italy; francesco.esposito4@unina.it (F.E.); teresa.rea@unina.it (T.R.); 2 Department of Promotion of Human Sciences and the Quality of Life, University of Study of Roma “San Raffaele”, via di Val Cannuta, 247–00166 Roma, Italy; salvatore.velotto@uniroma5.it; 3 Department of Science and Technology, Newton Consulting Srl, 80146 Napoli NA, Italy; tommasostasi@newtonconsulting.it; 4 Department of Agricultural Sciences, University of Naples “Federico II”, via Università, 100–80055 Portici, Naples, Italy

**Keywords:** margin of exposure, acrylamide, 2-propenamide, bromination, risk assessment

## Abstract

Acrylamide (AA) is a neo-formed toxic compound that develops in foods during cooking at temperatures above 120 °C. AA shows in vivo neurotoxic and carcinogenic effects, and it is potentially carcinogenic for humans. Its occurrence is common in baked food, such as bread and similar products. This study set out to analyze bread and sweets from the Italian market to evaluate the effects of the benchmark thresholds set by EU Regulation 2017/2158 and to ascertain the exposure of the Italian population to AA, across three age groups, through the consumption of baked products, according to the margin of exposure (MOE) approach. Two hundred samples were tested, and the content of AA ranged from 31 to 454 µg/kg for bread and products thereof and from 204 to 400 µg/kg for the sweets category. The exposure data did not show any neurotoxic health concern, whereas the MOE related to the carcinogenic endpoint is well below the minimum safety value of 10,000.

## 1. Introduction

Acrylamide (AA) is a food process toxicant, namely a compound that develops during cooking at temperatures above 120 °C, in foods with substantial levels of asparagine and reducing sugars, and it is not detected in boiled food [1,2,3], even though a sharp increase in its formation occurs at a temperature above 170 °C [4,5]. Its mechanism of formation mainly involves the Maillard reaction; hence, detectable levels of AA are common in baked foods, but it also occurs in fried potato, French fries, savory snacks, starchy extruded products, and coffee [6,7,8,9,10]. A huge amount of literature has focused on the toxic effects related to the ingestion of AA on the basis of the experimental evidence on rats and mice that demonstrates detrimental effects, such as neurotoxicity, genotoxicity, and developmental toxicity. However, according to the International Agency for Research on Cancer, since there is no epidemiological evidence of carcinogenicity on humans, AA was classified as potentially carcinogenic to humans (group 2A) [11,12]. Because of its recognized toxicity, AA has been studied by many researchers in a perspective of suitable mitigation measures to avoid its formation during the food processes [13,14,15,16,17,18]. As mentioned above, baked products show noticeable levels of AA and among them, bread and similar products could be a significant source of dietary exposure to AA for consumers due to the high quantity that is ingested every day by consumers. The formation of AA during the baking process of bread can be influenced by many factors, such as moisture content, pH, temperature, and time [19]. During the Maillard reaction, a lot of desired compounds develop, influencing and enhancing the taste, flavor, and color of the baked product, but due to the high temperature required for this process, the formation of AA in these products is an unavoidable drawback [20,21]. 

In a recent report, EFSA performed a risk characterization related to the occurrence of AA in processed food, evaluating literature data on new toxicological studies and the evidence of carcinogenicity exerted by AA, eventually providing a dietary exposure assessment to AA, based upon the margin of exposure (MOE) approach on neurotoxic and carcinogenic effects. This report confirmed the occurrence of AA in a high number of heat-processed foods, and the dietary exposure pointed out the potential increased risk of developing cancer in consumers of all age groups [6].

On the basis of this evidence, in 2017, the European Commission (EC) issued a regulation (EU Regulation 2017/2158) establishing mitigation measures and benchmark levels for the reduction in the presence of acrylamide in food [22]. According to this regulation, the levels of AA in wheat-based soft bread and crispbread should not exceed 50 and 350 µg/kg, respectively. The limit for biscuits and ice cream wafers is 300 µg/kg, whereas the threshold for other products (which applies to sweets) is 350 µg/kg.

Therefore, this study aimed to evaluate the levels of AA in different Italian baked products (bread and products thereof and sweets), to give an insight on the levels of AA after two years following the entry into force of the UE regulation 2017/2158 and in order to ascertain the dietary exposure of the population to this compound, through a deterministic approach based upon the MOE.

## 2. Results and Discussion

The levels of AA were above the limit of quantification (LOQ) in all samples, and the concentrations were in the range 31–454 µg/kg as regards bread and products thereof, whereas as far as sweets and biscuits are concerned, the concentrations varied from 204 to 400 µg/kg (Table 1).

From the data in this table, it is apparent that the mean and median levels of AA in the bread category were above the benchmark levels issued by EU regulation 2017/2158. Besides, no statistically significant difference emerged within the soft bread products whose means were instead significantly smaller than those observed in “Friselle” products (a kind of twice-baked bread produced with the same dough of soft bread). An impressive result to emerge in sweets is that most of this category shows AA levels below the EU benchmark limits, as well as some values at the 95th percentile that were detected in the samples. This result is evident, especially in butter cookies, “Babà” cake, and ice cream wafers and is somewhat surprising considering that the content of sugars in these products is high. However, AA formation is a complex mechanism related not only to the concentration of precursors but also to the time and temperature of the baking process and moisture percentage. The moisture, sugars, and salt percentages are displayed in Table 2 and Table 3 based on the recipes of each product that were gathered during the sampling, either for bread or sweets categories.

**Figure 1:**
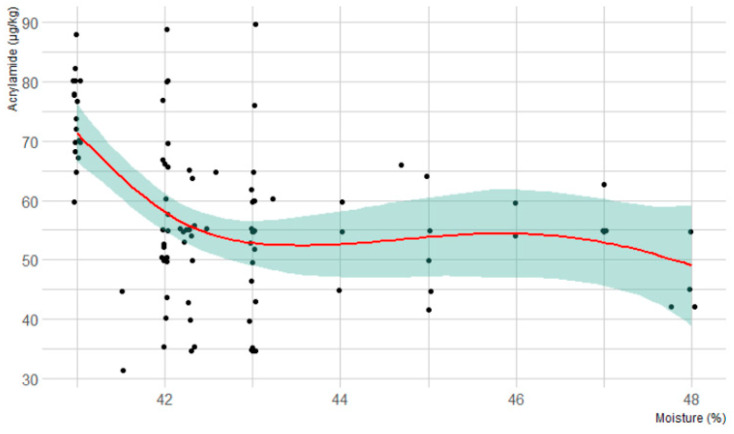
Curvilinear relationship between acrylamide levels and moisture content in refined flour bread and wholemeal bread and bread rolls (the light blue area indicates the 95 percent confidence interval).

As regards bread, bread rolls, and wholemeal bread, a slight negative correlation emerged between the final AA levels and the moisture percentage of the dough before the baking process (Spearman’s rank correlation ρ = −0.44, *p*-value < 0.001). As a matter of fact, Figure 1 shows a curvilinear relationship between the starting moisture percentage and the levels of AA in the finished product (Figure 1). According to this figure, the content of AA is characterized by a sharp increase below the 43% moisture percentage of the dough. Several studies reported that low-moisture triggers acrylamide formation, especially in starchy food, and this evidence might be explained by water evaporation that influences the temperature of the surface of the product during the baking process, thus delaying the formation of AA [23,24,25].

This trend was not statistically significant in the sweets category, instead for Spearman’s rank correlation, *p*-value > 0.280. A likely explanation for this might be that the starting moisture percentage and its effects on AA formation could be influenced by other ingredients. Mesías et al. (2020) suggested that the presence of egg, butter, or a high amount of sugar (greater than 20%) in the formulation seems to play a key effect in the final concentration of AA [26]. This finding seems to be corroborated by the AA levels detected in our work in butter biscuits, ice-cream wafers, and “Babà” cakes where, despite the highest percentage of sugars used in the recipe, no sample exceeded the benchmark threshold of Regulation 2017/2158 and showed the lowest levels in the sweets category.

As far as the bread category is concerned, the levels of AA reported in this study are lower than the data observed in toast and bread from the Slovenian market that ranged from 44.5 μg/kg to 246.1 μg/kg [4]. In contrast, they are consistent with results observed by Mojiska et al. (2010) and in Croatian wheat bread sampled between January 2015 and April 2018, whereas the levels of AA from the latter study related to bread samples collected after April 2018 are lower than our results and so are the data by EFSA (2015) and Roszko et al. (2020) concerning soft wheat bread products [6,19,27,28].

### Exposure Assessment

The dietary exposure was evaluated on bread and similar products, since accurate consumption rates about typical sweets are lacking, and hence, any exposure estimation on this category would have been biased.

Higher exposure values occurred among adolescents either median or consumers at the 95th percentile, and this was due either to the dietary habits or the lower body weight of younger consumers (Table 4 and Table 5). Besides, adolescents also showed the lowest exposure value as regards the product wholemeal Friselle: this was due to the fact that the consumption of this product is not common among young individuals. In the case of median elderly consumers, their exposure through bread and product thereof is always higher than adults, due to higher consumption rates, also considering that the same body weight was used for both age groups. Quite the opposite applies in consumers at the 95th percentile, where the consumption rates of adults are higher or comparable to the data of the elderly, except for “Friselle”, which is made of refined flour.

The bread rolls were excluded from the daily intake assessment as the consumption data of bread and bread rolls are reported as a whole in the comprehensive database.

In accordance with the present study, Mencin et al. (2020) reported a daily exposure of an adult population to AA through the ingestion of bread ranging from 70 to 380 ng/kg bw/day (mean: 210 ng/kg bw/day) [4]. However, the exposure data obtained in this study are far above those observed by Andačić et al. (2020) [19].

**Figure 2:**
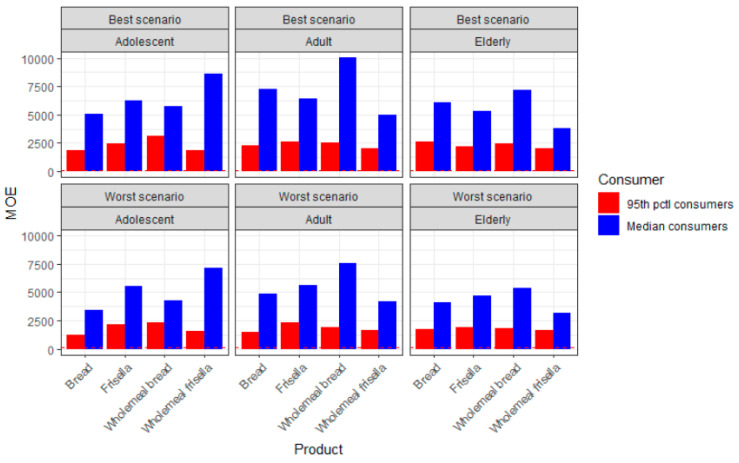
Margins of exposure (MOE) values for neurotoxicity of acrylamide (AA) through the consumption of bread and products thereof, across different consumers and according to best and worst scenarios (The y-intercept at 125 is a threshold, as it accounts for the minimum value at which the neurotoxic MOE is considered of low concern).

**Figure 3:**
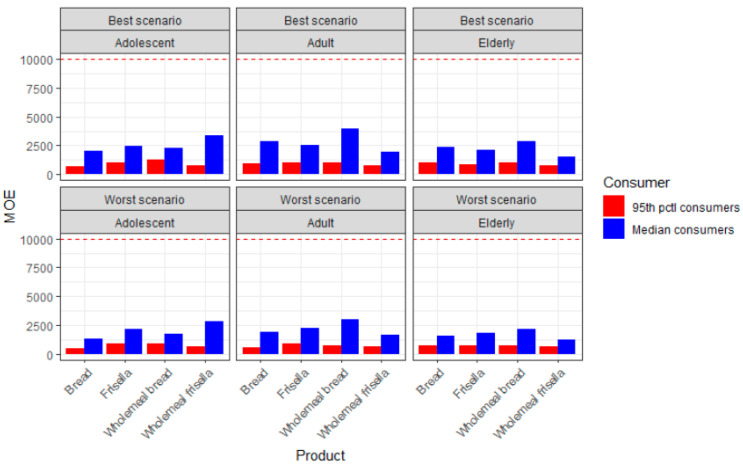
Margins of exposure (MOE) values for the carcinogenicity of acrylamide (AA) through the consumption of bread and products thereof, across different consumers and according to best and worst scenarios (the y-intercept at 10,000 is a threshold, as it accounts for the minimum value at which the carcinogenic MOE is considered of low concern).

The MOE related to neurotoxic endpoints varied from 1225 to 10,033, and according to EFSA, a value of 125 or above is considered of no concern as regards neurotoxicity. According to our results, the risk of neurotoxicity is of low health concern (Figure 2). By contrast, the MOE related to carcinogenicity is based on a BMDL_10_ of 0.17 mg/kg bw/day, and a value greater than 10,000 is considered of low concern. The values observed in the present study related to the carcinogenic effects of AA were in the range 484–3967, and this finding entails a health concern for the population of all age groups that were considered in this study (Figure 3).

Taken together, our data on risk characterization are in line with those by the EFSA; however, it is worth highlighting that the impact of uncertainties in dietary exposure to AA could play a not insignificant role in the overall risk assessment. The main uncertainties are related to the carcinogenic outcomes based on the Harderian gland (not reproducible in humans) as well as the inconsistency in the epidemiological studies of associations between dietary exposure and carcinogenicity [6]. Therefore, further epidemiological studies are certainly recommended in order to ascertain possible associations between dietary AA ingestion and risk of cancers, mainly focusing on those carcinogenic endpoints reproducible in humans.

## 3. Materials and Methods

### 3.1. Sampling

A total of 200 samples were collected at local manufacturers of Campania region (Italy), just after the baking process. The samples consisted of baked products—namely, bread, bread rolls, and whole wheat bread, “Friselle” and wholemeal “Friselle” (a traditional twice-baked bread commonly used in Italy—and traditional Italian sweets—“Babà” cake, ice cream wafers, “Frolla” cake, and croissants. The products were stored at room temperature after the sampling and underwent analysis within 24 h. The recipes of these products were obtained by the manufacturers in order to evaluate the possible influence of ingredients on the final level of AA.

### 3.2. Reagents and Equipment

Acetonitrile, ethyl acetate, n-hexane (all GC grade), and sodium chloride (analytical grade) were supplied by Merck KGaA, (Darmstadt, Germany); AA (purity ≥ 99.9%), and the internal standard (IS) AA^−13^C_3_, hydrobromic acid (48% *w*/*w* aq.), sodium thiosulfate pentahydrate, potassium bromide (both analytical grade) were purchased from Sigma Aldrich (St. Louis, MO, USA); bulk C18 (size 125 Å 55–105 µm) supplied by Waters (Milford, MA, USA); saturated bromine solution was supplied by Titolchimica (Pontecchio Polesine, RO, Italy). Agilent 7890A GC-MS system coupled to an Agilent 5975C mass selective detector (MSD) (Agilent Technologies, Santa Clara, CA, USA); the column was a Restek Rxi^®^-XLB GC (proprietary phase; length × I.D.: 30 m × 0.25 mm; df: 0.25 µm) (Restek, Bellefonte, PA, USA).

### 3.3. Preparation of the Analytical Standards

A stock standard solution of 1 g/L was prepared dissolving 50 mg of AA in acetonitrile to a total volume of 50 mL and a working solution of 10 mg/L. The standards were processed through the same procedure of the samples, starting from aliquots between 1000 and 12.5 µL of working solution (corresponding, respectively, to a range of 1000–12.5 ng of AA and equivalent to a range of 25–2000 µg/kg in the samples). A working solution of 10 mg/L of IS was also prepared.

### 3.4. Extraction

The detection of AA in selected samples was performed by adapting the UNI CEN/TS 17083:2017 method and procedures used by Fernandes and Soares (2007) and Esposito et al. (2017) [8,29,30], applying a matrix solid-phase dispersion (MSPD) extraction, followed by bromination of aqueous extracts. Briefly, 0.5 g of ground sample was added to 1.5 g of C18 bulk sorbent and well mixed in a glass mortar. Then, the mix was packed into an empty polypropylene cartridge and a polyethylene frit was put at the bottom and onto the top of the packed mix. The cartridge was placed onto a vacuum manifold and the sample was defatted with 10 mL of n-hexane at a flow rate of about 5 mL/min, and finally, the cartridge was dried by applying a vacuum for 2 min. AA was eluted by adding 5 mL of water holding the flow for 5 min to permit a better extraction of the compound (this step was repeated twice). Finally, the extract was collected in a glass vial and underwent bromination, according to the procedure described in the next section. Each sample was analyzed in triplicate.

### 3.5. Bromination

1 g of KBr was added to the aqueous extracts previously collected, and the solution was acidified until pH 1–3 with 1–2 drops of HBr (48% *w*/*w*). Then, 2 mL of saturated bromine solution were added to the aqueous samples that were stored in an ice bath for 2 h in the dark. 4–7 drops of sodium thiosulfate 1 M solution were added to the sample until the yellow color of the solution turned transparent. Then, 4 g of NaCl were added to the solution and the brominated derivative was extracted with 10 mL ethyl acetate: n-hexane mixture (4:1 *v*/*v*), pooling the upper layers; this step was repeated one more time with 5 mL of the ethyl acetate: n-hexane mixture. The collected phase was dried to 2 mL under a stream of nitrogen at 40 °C, and half a spatula of anhydrous sodium sulfate was added. The extract was centrifuged at 4000 rpm for 5 min, and the extract was transferred to a graduated glass vial and dried up to 0.5 mL under nitrogen before injection.

### 3.6. GC-MS Detection

The quantification of AA was performed on a GC-MS system according to the following operating conditions: the carrier gas was helium at a flow rate of 1.0 mL/min; the volume of injected sample was 1 µL in pulsed splitless mode; the temperature of injector was held at 280 °C. After the injection, the column was held at 85 °C for 1 min, then the ramp rate was 15 °C/min until 280 °C, holding this temperature for 16 min for a total run time of 30 min. The transfer line of the GC-MS interface was at 280 °C. The retention time of the target compound and the internal standard ^13^C_3_-AA was 6.90 min. The mass spectrometer operated in electron ionization mode (EI), with a collision energy of 70 eV. The analysis was performed in selected ion monitoring (SIM) mode, and the selected ions were *m*/*z* 150 (quantifier ion) and *m*/*z* 152, 108, and 106 (qualifier ions) for the derivative 2,3-dibromopropionamide and *m*/*z* 153 (quantifier ion) and *m*/*z* 155 and 109 (qualifier ions) for the IS.

### 3.7. Method Performance

The calibration curve was plotted as the peak area ratio of 2,3-dibromopropionamide to the IS versus the concentration. The limit of detection (LOD) and the limit of quantification (LOQ) were calculated using the standard deviation of the response (σ) and the slope of the calibration curve (S), according to the following formulas:

LOD = 3.3 σ/S(1)

LOQ = 3 LOD(2)

LOD and LOQ values were 8 µg/kg and 25 µg/kg, respectively. Recoveries were tested spiking three aliquots of ground sample with AA standard and IS at the following concentrations: 200 µg/kg + 500 µg/kg (IS), 500 µg/kg + 500 µg/kg (IS), and 1000 µg/kg + 500 µg/kg (IS); for each concentration six samples were analyzed, and the recoveries ranged from 92% to 97%. The precision of the method was calculated assessing the intra-day and inter-day repeatability through the injection of the three samples used for the recoveries, five times a day for seven consecutive days. The intra-day repeatability expressed as the relative standard deviation (RSD) ranged from 4 to 7%, and the inter-day repeatability was below 7%.

### 3.8. Data Analysis

Descriptive and inferential data analysis and graph processing were performed using R Software version 3.5.3 and the following packages: ggplot2, readxl, and hrbrthemes [31,32,33,34].

### 3.9. Exposure Assessment and MOE Evaluation

The dietary exposure assessment involved three categories grouped by age (adolescent, adult, and elderly). The median and the 95th percentile (pctl) daily consumption for each food category with the corresponding concentrations of AA occurred in the samples (median and 95th percentile levels) were considered, and their product was divided by the body weight (bw) of each individual according to the age groups. On this basis, the chronic exposure to AA was calculated according to the formula:

DI = (C × Q)/BW(3)

DI = daily intake of acrylamide (ng/kg bw/day).
C = median and 95th percentile concentration of acrylamide detected in the samples (ng/g).
Q = individual food daily consumption of population within different age groups and for median and 95th pctl consumers (g/day).
BW = individual body weight (kg bw).

The concentrations of AA (C) that were used for the calculation of the daily intake were the median and the values at the 95th pctl detected in the samples. The individual food daily consumptions (Q) of Italian people were gathered from the Comprehensive European Food Consumption Database by the EFSA, considering an exposure hierarchy level 7 and from the study by Leclercq et al. (2009): the median and the values at the 95th pctl for median consumers and the analog values for consumers at the 95th pctl were used for the calculation. The body weight (BW) of consumers was drawn from anthropometric data of Italian people within the three age groups, according to Leclercq et al. (2009) [35]. A total of two scenarios were considered, for each age group: best scenario (BS) was where the individual food consumption (Q) were the median and the consumers at the 95th percentile and the concentrations of AA (C) were the median value detected in the samples; worst scenario (WS) was where C was the concentrations values at the 95th percentile that were detected in the samples. Finally, the chronic intake values were used to perform a risk characterization of neurotoxic and carcinogenic effects of AA through the MOE, according to the following formula:

MOE = BMD**L**_10_/DI (4)

MOE = margin of exposure (dimensionless).
BMDL_10_ = benchmark dose lower confidence limit for a benchmark response of 10% (mg/kg bw/day).
DI = daily intake of acrylamide previously calculated (mg/kg bw/day).

The values of BMDL_10_ considered for the estimation were 0.43 mg/kg bw/day and 0.17 mg/kg bw/day derived from experimental evidence of peripheral nerve axonal degeneration in rats and adenocarcinomas of the harderian gland in mice [6].

## 4. Conclusions

To sum up, it was observed that after two years following the entry into force of EU regulation 2017/2158, a high percentage of bread and similar products on the Italian market could exceed the benchmark levels of AA. Therefore, undertaking mitigation measures to reduce AA plays a crucial role in raising MOE values to safer levels, especially for bread that could be an essential contributor to the daily intake of AA among the Italian population. For this purpose, the correct choice of time and temperature of the baking process and the use of dough with a higher percentage of moisture could be an effective mitigation measure, as far as soft bread is concerned. Finally, it seems necessary to better understand the role of sugars, eggs, butter, and other ingredients in the formation mechanism of AA during the production of biscuits and other sweets, to improve the recipes from the perspective of new mitigation approaches.

## Figures and Tables

**Table 1:** Summary statistics of levels of acrylamide bread and sweets.

Category	Product	Acrylamide (µg/kg)				Benchmark Levels According to EU 2017/2158 (µg/kg)
		Mean ± SD	Median	95th Percentile	Min–Max	
Bread (*n* = 102)	Bread	57 ± 18 ^a^	55	82	31–90	50
	Bread rolls	52 ± 8 ^a^	55	64	42–67	50
	Wholemeal bread	61 ± 10 ^a^	60	80	44–88	50
	Friselle	358 ± 36 ^b,c^	353	403	306–454	350
	Wholemeal Friselle	384 ± 37 ^b^	375	450	328–450	350
Sweets (*n* = 98)	Butter cookies	310 ± 36 ^d,e^	330	346	249–350	350
	“Babà” cake	292 ± 62 ^e^	278	391	207–400	300
	Ice cream wafers	337 ± 5 ^c,d^	336	344	330–346	350
	“Frolla” cake	362 ± 11 ^b,c^	356	380	350–380	300
	Croissants	313 ± 55 ^d,e^	330	382	204–396	300

^a,b,c,d,e^ Mean values with the same letter for each product are not statistically different (one-way ANOVA with Tukey’s HSD post-hoc test *p* < 0.01).

**Table 2:** Moisture, sugar, and salt percentage in each product of bread category.

Product	Moisture (%)	Sugars (%)	Salt (%)
Bread (*n* = 22)	41–43	1.0–1.2	1.6–1.8
Bread rolls (*n* = 22)	42–48	1.0–1.2	1.5–1.7
Wholemeal bread (*n* = 22)	41–45	1.0–1.2	1.6–1.8
Friselle (*n* = 18)	39–42	1.1–1.3	1.7–1.9
Wholemeal Friselle (*n* = 18)	35–40	1.2–1.4	1.5–1.8

**Table 3:** Moisture, sugar, and salt percentage in each product of sweets category.

Product	Moisture (%)	Sugars (%)	Salt (%)
Butter cookies (*n* = 20)	14–18	36–39	0.5–0.8
“Babà” cake (*n* = 20)	43–46	5–6	0.0–0.1
Ice cream wafers (*n* = 20)	34–35	22–24	0.0–0.1
“Frolla” cake (*n* = 18)	30–32	3–5	0.0–0.1
Croissants (*n* = 20)	36–38	10–12	1.0–1.1

**Table 4:** Daily intake of acrylamide (AA) of median consumers according to the two scenarios considered in this study (BS = best scenario; WS = worst scenario).

Product	Daily Exposure (ng/kg bw/day) (BS–WS)		
	Adolescents	Adults	Elderly
Bread	84–125	59–88	71–105
Wholemeal bread	75–100	43–57	60–80
Friselle	68–78	67–77	81–92
Wholemeal Friselle	50–70	86–103	114–137

**Table 5:** Daily intake of acrylamide (AA) of consumers at the 95th percentile according to the two scenarios considered in this study (BS = best scenario; WS = worst scenario).

Product	Daily Exposure (ng/kg bw/day) (BS–WS)		
	Adolescents	Adults	Elderly
Bread	236–351	189–281	165–246
Wholemeal bread	137–182	171–229	174–232
Friselle	173–197	161–184	197–225
Wholemeal Friselle	230–276	214–257	213–255
